# Emergency medical preparedness and response for mass gatherings: Papua New Guinea emergency medical team's experience during Pope Francis's 2024 visit

**DOI:** 10.5365/wpsar.2026.17.2.1233

**Published:** 2026-04-23

**Authors:** Ulysses Oli, Garry G Nou, Rose Hosea, Kelvin Konigala

**Affiliations:** a Papua New Guinea Emergency Medical Team, Port Moresby, Papua New Guinea.; b Port Moresby General Hospital, Port Moresby, Papua New Guinea.; c Department of Health, Port Moresby, Papua New Guinea.

## Abstract

**Problem:**

Papua New Guinea has hosted several mass gatherings in recent years. Yet, there is limited data on the involvement of emergency medical teams in these events, let alone in other low- and middle-income countries. Prior to Pope Francis’s state visit in September 2024, the national emergency medical team (EMT) had not simulated its updated clinical patient flow systems.

**Context:**

During Pope Francis's visit, an estimated 70 000 attendees gathered at the national stadium. Using Arbon’s mass gathering model, the patient presentation rate was calculated to be 330 over 2 days, or 4.7 per 1000, posing a significant challenge to local health-care capacity.

**Action:**

The National Department of Health mobilized its EMT to lead clinical operations in collaboration with the National St. John Ambulance service. Planning spanned 6 weeks and involved provincial partners.

**Outcome:**

Over 2 days, 257 patients, or 3.7 per 1000, were managed using a hub-and-spoke model. First-aid stations operated by the St. John Ambulance service treated 200 patients for dehydration and headaches, while the Advanced Casualty Management Centre handled 57 cases of heat-related illnesses and chronic condition exacerbations.

**Discussion:**

The deployment of the EMT showcased its capability and credibility, setting a national milestone and providing a feasible model for mass-gathering preparedness and response in low- and middle-income countries in the World Health Organization’s Western Pacific Region. This report highlights not only the importance of multipartner collaboration in such preparedness, providing baseline data for low-resource settings, but also the scalability of rehabilitation services and the Interagency Integrated Triage Tool as frameworks for future deployments.

## PROBLEM

Mass gatherings (MGs) can significantly strain the resources of host communities, increasing the risk of injuries, illnesses and catastrophic incidents. (*1*) MGs, including cultural, religious, political and sporting events, often lead to higher injury and illness rates among the attendees than in the general population. (*2*) Thorough planning is mandatory for risk mitigation and timely access to health care. (*2*) The principles of MG medical care include rapid patient access and triage, effective stabilization and transport of critically ill or injured patients, and onsite management of minor injuries and illnesses. (*2*)

In Papua New Guinea (PNG), past events like the 15th Pacific Games (2015) and the 26th Asia–Pacific Economic Cooperation Summit (2018) demonstrated the importance of a multisector approach and an integrated emergency care system in managing such large-scale events. (*3*, *4*) However, data on national emergency medical team (EMT) involvement in MGs, particularly in low- and middle-income countries (LMICs) in the Pacific, remain scarce. (*5*) Most regional studies focus on disaster or pandemic responses, with limited attention to MGs. (*6*-*10*) Although the PNG EMT had achieved key milestones, including deployments during the COVID-19 surge, member training and procurement of an EMT cache, it had not deployed for an MG until the state visit of Pope Francis in 2024. (*7*, *8*, *11*) This report highlights the first deployment of the PNG EMT for an MG, documenting the strengths, challenges and strategies for improving operational readiness in resource-limited settings.

## CONTEXT

Pope Francis’s state visit to PNG in September 2024 tested the country’s emergency medical response capabilities. An estimated 70 000 attendees were expected at the Sir John Guise Stadium in Port Moresby for the primary event, a Holy Mass, with an additional gathering in West Sepik Province. (*12*) Due to the stadium's limited capacity of 35 000 people, events were dispersed across multiple venues to accommodate the crowd, increasing the complexity of medical planning, as first-aid stations and response teams needed to cover multiple sites.

The MG presented several health risks, informed by prior regional and global experiences and World Health Organization (WHO) guidelines. (*1*, *5*) Prolonged outdoor exposure posed risks of heat-related illnesses and dehydration, worsened by high temperatures and a lack of shade in the outdoor stadium. The high attendance raised concerns about the potential for a mass casualty incident, such as a crowd crush or structural failure, particularly given the stadium’s capacity constraints, which necessitated crowd control measures and enhanced triage capacity. (*2*) These concerns were considered during risk assessments, prompting the use of a hub-and-spoke model and contingency plans for rapid escalation to nearby hospitals. Additionally, the large crowd size increased the risk of infectious disease transmission, such as respiratory illnesses, prompting public health measures such as mask distribution.

The PNG National Department of Health (NDOH) and its EMT worked closely with the National St. John Ambulance (NSJA) service, which was instrumental in developing a comprehensive preparedness plan in collaboration with other key partners. These partners included the country’s top-tier public referral hospital, Port Moresby General Hospital (PMGH), as well as the National Capital District Provincial Health Authority (NCDPHA) and the West Sepik Province Health Authority (WSPHA). External partners included the National Events Secretariat, the Ministry of Foreign Affairs, the PNG Defence Force and the West Sepik Provincial Emergency Operation Centre (WSPEOC). These partners played crucial roles in the operational framework. Internal partners included organizations directly involved in health care, such as PMGH and two provincial hospitals in the areas where the Pope's visit took place. These comprised Gerehu General Hospital under the authority of NCDPHA, and Vanimo General Hospital under WSPHA. External partners involved organizations in logistics, defence and emergency response, as well as government agencies responsible for national coordination and diplomacy, including the Ministry of Foreign Affairs and the National Events Secretariat. This multipartner approach addressed the identified risks and ensured a coordinated response across dispersed venues.

## ACTION

### Overall planning and methods

The NDOH was the strategic lead, coordinating nationwide planning with various internal and external partners. Planning commenced in February 2024 and was divided into two phases.

In Phase 1, a multipartner, coordinated response was outlined in an overarching Health Operations Plan (Papal visit health operations plan. Port Moresby: National Department of Health, National St. John Ambulance, unpublished data, 2024) based on *Emergency response framework (ERF): internal WHO procedures*. (*13*) NSJA was instrumental in the preparation efforts, representing health stakeholders in inter-agency meetings and contributing to pre-hospital care, logistics, critical care and aeromedical support. PMGH provided clinical, pharmaceutical and rehabilitation staff, most of whom were trained under the PNG EMT programme. NCDPHA oversaw the public health response, deploying rapid response teams. WSPHA and the WSPEOC supported NDOH during the Pope's visit to West Sepik Province, which is 946 km from Port Moresby. International EMTs were on standby for crisis-level surge capacity.

Phase 2 involved simulations and walkthroughs conducted with all response teams in the weeks leading up to the event. Arbon’s mass gathering model, a predictive tool used to estimate patient presentation rate (PPR) based on crowd size, event characteristics and environmental factors, was applied to forecast service demand. This model estimated a PPR of 330 over 2 days, or 4.7 patients per 1000 attendees daily. (*14*) This projection informed staffing, equipment allocation and clinical capacity planning and highlighted the potential strain on local health-care resources. (*2*)

### Clinical planning for Type 1 EMT

**Fig. 1:**
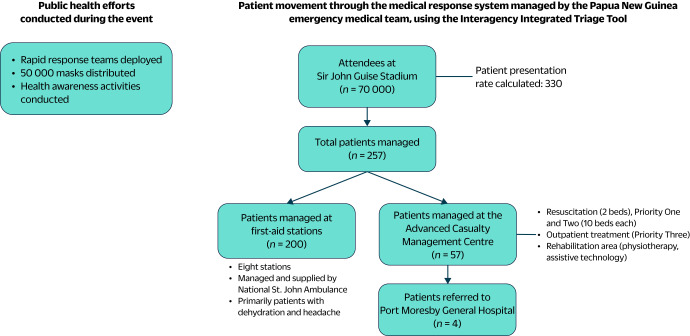
Patient movement through the medical response system and concurrent public health activities during the papal visit to Papua New Guinea, 2024

The PNG EMT, a Type 1 EMT focused on outpatient care, planned for the calculated PPR of 330 over 2 days, providing onsite clinical and rehabilitation services using established patient pathways. EMT members used the Interagency Integrated Triage Tool (IITT) (*15*) to improve patient flow and include rehabilitation for musculoskeletal injuries. NSJA supplied mobile medical kits, pharmaceuticals and first-aid teams at eight stations to address issues such as dehydration and headaches (**Fig. 1**).

[**insert** Figure 1]

**Fig. 2:**
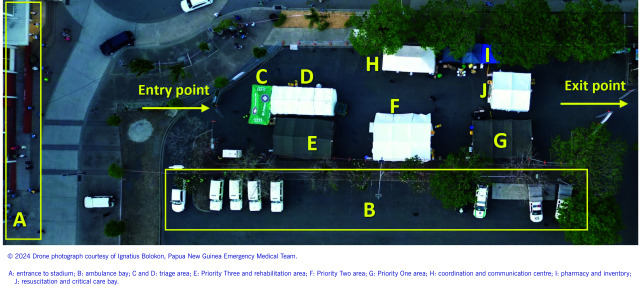
Layout of the Advanced Casualty Management Centre and its various stations during the papal visit to Papua New Guinea, 2024

Despite its role in providing outpatient care, the diverse skills of PNG EMT members, including those of emergency physicians, surgeons and senior non-specialist doctors, enabled the establishment of a temporary resuscitation and critical care bay within the Advanced Casualty Management Centre (ACMC) – the primary onsite medical hub (**Fig. 2**). Equipped for advanced procedures like intubation, electrical cardioversion and defibrillation, it ensured readiness for critical cases. The small team required close collaboration with NSJA staff, who provided standby support to enhance critical care capacity.

[**insert** Figure 2]

NSJA coordinated contingency plans and arranged the transfer of patients: low-priority patients to Gerehu General Hospital and high-priority patients to PMGH. They also supported Pope Francis’s trip to West Sepik Province with an aeromedical escort. Two-week pre-event simulations included scenario-based training on the IITT and patient flow, ensuring staff proficiency in triaging and transitions between first-aid stations and the ACMC.

Rehabilitation services were added to the clinical plan, with physiotherapists trained in acupuncture and assistive technology like wheelchairs and walking aids sent to the ACMC to address mobility needs. The plan was supported by a logistics system that ensured sufficient supplies of oral rehydration salts, pain relievers and rehabilitation equipment, tailored to resource limitations common in LMICs. This strategy enabled the PNG EMT to manage outpatients and standby for critical care during the papal visit.

### Setting

Patient flow was streamlined using a hub-and-spoke model, with first-aid stations passing patients to the ACMC (**Fig. 1**). Located in the north-west car park of the stadium where the Holy Mass was held (**Fig. 2**), the ACMC operated under a three-colour triage system based on the IITT. (*15*) The system facilitated efficient patient flow and the integration of physical rehabilitation into the ACMC by categorizing patients into Priority One (immediate, life-threatening conditions requiring resuscitation), Priority Two (urgent conditions requiring early intervention) and Priority Three (non-urgent or minor conditions). (*15*)

The ACMC spanned 700 m^2^ (14 × 50 m) with a 23-bed capacity, comprising:

- 2-bed resuscitation ward;
- 10-bed Priority One ward;
- 10-bed Priority Two ward; and
- 1-bed examination/rehabilitation area in the Priority Three ward.

### Staff

The PNG EMT lead and the NSJA Chief Medical Officer headed operations as Health and Deputy Health Commander, respectively. Their team included an incident manager, public health commander, logistics commander, and sector commanders for NCDPHA and WSPEOC.

The NSJA deployed 50 personnel, including emergency physicians, paramedics, ambulance officers, critical care nurses, pharmacists and logistics officers. PMGH contributed 17 staff members, 14 of whom were trained by the PNG EMT, including emergency doctors, nurses, physiotherapists, a critical care anaesthetist, an orthopaedic surgeon, a health extension officer, an anaesthetic scientific officer, a pharmacist, and logistics and data officers. A dedicated aeromedical team, consisting of a critical care anaesthetist and critical care nurse, provided escort services for Pope Francis to West Sepik Province.

### Data collection

Members of the PNG EMT collected patient data on standardized paper-based triage forms based on the IITT. A designated logistics officer compiled data from the first-aid stations and the ACMC, which were aggregated daily into a centralized electronic log post-event, with supervisory cross-checks to ensure accuracy. This process enabled real-time tracking of patient presentations and outcomes, despite resource constraints typical in an LMIC setting.

## OUTCOME

### Patient care and results

The onsite first-aid stations and the ACMC aided in surge capacity, thus reducing the patient load in Port Moresby's resource-constrained hospitals. Patient care began at one of eight strategically placed first-aid stations inside the stadium. Patients requiring advanced care were redirected to the ACMC, where the IITT guided triage and treatment. Referrals for specialized care were directed to PMGH.

Over the 2-day event, 257 patients, or 3.7 per 1000, received care. First-aid stations managed 200 cases with primarily dehydration and headaches. The ACMC treated 57 patients, comprising 11 Priority Two and 46 Priority Three patients. These included 35 males, 22 females, two children under 5 years and eight patients over 50 years (**Table 1**). Four patients required urgent referral to PMGH:

**Table 1:** General characteristics of patients who visited the Advanced Casualty Management Centre for medical review during the papal visit in Papua New Guinea, 2024

Characteristic	Day 1	Day 2	Total
**Subtotal**	**28**	**29**	**57**
**Sex**			
**Male**	**17 (61)**	**18 (62)**	**35 (61)**
**Female**	**11 (39)**	**11 (38)**	**22 (39)**
**Age range, years**			
**Median age**	**36**	**42**	**35**
**0–5**	**2 (7)**	**0 (0)**	**2 (4)**
**6–15**	**3 (11)**	**0 (0)**	**3 (5)**
**15–50**	**19 (68)**	**25 (86)**	**44 (77)**
** > 50**	**4 (14)**	**4 (14)**	**8 (14)**
**Nationality**			
**Papua New Guinea**	**27 (96)**	**29 (100)**	**56 (98)**
**Expatriate**	**1 (4)**	**0 (0)**	**1 (2)**

• Values are *n*(%) unless otherwise indicated.

- a 6-month-old infant with acute febrile illness and signs of meningism;
- a 12-year-old boy with facial cellulitis;
- a 2-year-old male with acute bronchiolitis; and
- a 40-year-old male with clinical pneumonia.

The most common presentations at the ACMC were acute outpatient conditions (17 cases), exacerbations of chronic illnesses (14 cases) and heat-related illnesses (12 cases). Additionally, 16 patients required physical rehabilitation services. They were managed onsite by experienced physiotherapists trained in acupuncture. Assistive technology, particularly wheelchairs and walking support, was provided to patients who required it.

Public health efforts complemented clinical care. The public health team distributed over 50 000 face masks and conducted health-awareness activities.

## Discussion

This deployment of the PNG EMT during Pope Francis's visit marked a significant milestone in its first-ever response to an MG event, setting a precedent for similar medical responses in PNG and the broader WHO Western Pacific Region. By effectively integrating rehabilitation services and using the IITT, this operation provides a valuable model for resource-limited settings facing such challenging situations.

Two key observations highlight the significance of this deployment:

- Rehabilitation services: Rehabilitation services played a critical role in managing musculoskeletal injuries, helping patients with mobility limitations recover and providing assistive technologies, such as wheelchairs, during the deployment. This highlights the importance of integrating rehabilitation into MG planning that aligns with WHO's guidelines for comprehensive health care during such events. (*16*)
- IITT system: The IITT system facilitated efficient patient flow and prioritization of care, even in the context of disparate staff familiarity. Its structure streamlined onsite management, reinforcing the importance of standardized triage protocols in MGs, as supported by multiple studies. (*17*)

While this deployment was largely successful, several limitations should be considered. PNG EMT’s first MG response lacked historical data for accurate caseload prediction. Arbon’s model offered a framework, but it had not been validated locally, highlighting the need for context-specific data. Some staff’s unfamiliarity with the IITT led to triage errors, such as the misclassification of some Priority Three patients as Priority Two, which caused delays and incomplete IITT form completion and reporting. Targeted remedial training, including IITT workshops and simulation drills with realistic MG conditions, could improve staff proficiency. Incorporating these into capacity-building programmes, possibly via WHO EMT guidelines, would enhance future MG readiness in LMICs. Finally, limited resources may necessitate partner collaborations.

Despite these limitations, the operation demonstrated notable strengths. The diverse clinical expertise of the team ensured comprehensive patient care, and the efficient referral process enabled seamless access to higher levels of care when necessary. Strong collaboration between the PNG EMT, NSJA and other partners was key to effective coordination and resource management, emphasizing the importance of interagency communication in successful MG preparedness and response.

The findings from this deployment are consistent with the expected trends in MG medical responses. (*2*, *14*) The PPR was slightly lower than predicted by Arbon's model (3.7 vs 4.7 per 1000), potentially due to an underreporting by the satellite teams managing patients or due to timely public health awareness efforts. The prevalence of heat-related illnesses and the exacerbation of chronic conditions was consistent with expected presentations during MGs, particularly those involving prolonged sun exposure.

This experience highlights several opportunities for future research and improvement. The validation of Arbon's model for the PNG context is crucial in refining planning and resource allocation. Further research could focus on the influence of specific environmental factors, such as temperature, humidity and rainfall, on patient presentations during MGs in PNG. Additionally, valuable insights for optimizing future deployments could be gathered by evaluating the integration of rehabilitation services into MG medical teams and by collecting data on team members’ feedback.

By addressing these areas and building on the lessons learned, PNG could enhance its capacity to deliver comprehensive health care during MGs, ensuring the safety and well-being of local communities and visitors.

### Conclusions and recommendations

This report highlights the value of involving a national EMT in MG events and emphasizes the importance of multipartner collaboration in a low-resource setting. It establishes baseline data for MG medical response in PNG, which other similar settings in the WHO Western Pacific Region could utilize. Key findings include integrating rehabilitation services and using the IITT system, which is crucial for patient care. We recommend improving triage training, enhancing data collection and establishing pre-deployment training for MGs to address identified gaps. This clinical response model proved effective during the papal visit and could be adapted by EMTs in other low-resource settings.
